# Diagnostic Accuracy of the Triglyceride and Glucose Index for Insulin Resistance: A Systematic Review

**DOI:** 10.1155/2020/4678526

**Published:** 2020-03-10

**Authors:** Adriana Sánchez-García, René Rodríguez-Gutiérrez, Leonardo Mancillas-Adame, Victoria González-Nava, Alejandro Díaz González-Colmenero, Ricardo Cesar Solis, Neri Alejandro Álvarez-Villalobos, José Gerardo González-González

**Affiliations:** ^1^Universidad Autonoma de Nuevo Leon, Facultad de Medicina y Hospital Universitario “Dr. Jose Eleuterio Gonzalez”, Endocrinology Division, Av. Madero y Gonzalitos S/n, Mitras Centro, Monterrey 64460, Nuevo León, Mexico; ^2^Universidad Autonoma de Nuevo Leon, Plataforma INVEST Medicina UANL-KER Unit Mayo Clinic (KER Unit México), Av. Madero y Gonzalitos S/n, Mitras Centro, Monterrey 64460, Nuevo León, Mexico; ^3^Knowledge and Evaluation Research Unit in Endocrinology, Mayo Clinic, Rochester 55905, MN, USA; ^4^Universidad Autonoma de Nuevo Leon, Facultad de Medicina y Hospital Universitario“Dr. Jose Eleuterio Gonzalez”, Research Unit, Av. Madero y Gonzalitos S/n, Mitras Centro, Monterrey 64460, Nuevo León, Mexico

## Abstract

*Introduction*. The triglyceride and glucose (TyG) index has been described as a biochemical marker of insulin resistance (IR); however, its diagnostic accuracy remains uncertain.

**Objective:**

To summarize the evidence assessing the diagnostic accuracy of the TyG index regarding IR.

**Methods:**

A comprehensive search in MEDLINE, EMBASE, Web of Science, and Scopus was performed without any language restriction. Studies assessing the diagnostic accuracy of the TyG index against the hyperinsulinemic-euglycemic clamp (HIEC) or any other IR biochemical were assessed independently and in duplicate. Diagnostic accuracy measures (sensitivity, specificity, positive predictive value, negative predictive value, and likelihood ratios) were extracted independently and in duplicate. The QUADAS-2 tool was used to assess the risk of bias of independent studies.

**Results:**

We identified 15 eligible studies with 69,922 participants and an overall quality of low to moderate. The TyG index was evaluated by HIEC and HOMA as reference tests. The highest achieved sensitivity was 96% using HIEC, and the highest specificity was of 99% using HOMA-IR, with a cutoff value of 4.68. AUC values varied from 0.59 to 0.88. Cutoff values for IR were variable between studies, limiting its comparability.

**Conclusion:**

In this systematic review, we found moderate-to-low quality evidence about the usefulness of the TyG index as a surrogate biochemical marker of IR. Due to the lack of a standardized IR definition and heterogeneity between studies, further validation and standardized cutoff values are needed to be used in clinical practice.

## 1. Introduction

Insulin resistance (IR) is one of the first metabolic abnormalities leading to the development of type 2 diabetes, and it is known to be a key mediator of its pathogenesis [1]. The hyperinsulinemic-euglycemic clamp (HIEC) is considered the current gold standard to determine IR [2]; however, it is a complicated and time-consuming method with limited applicability to research settings. As an alternative strategy, surrogate markers derived from faster and less costly biochemical measurements have been proposed [2, 3]. To date, several IR surrogate markers such as HOMA-IR, TGC/HDL, QUICKI, and the McAuley index have been studied with different sensitivities and specificities for IR [4, 5].

Lately, the triglyceride and glucose index (TyG index) has become an attractive option due to the highly available and inexpensive biochemical markers needed for its calculation [6, 7]. It is derived from fasting plasma glucose and fasting triglyceride levels. The diagnostic accuracy of the TyG index in identifying IR using the HIEC and HOMA-IR as reference standards has been tested in several studies. However, the lack of consistency in their findings limits its generalizability and utility as a diagnostic marker of IR. Therefore, we sought to conduct a systematic review to assess the body of evidence regarding the diagnostic accuracy of the TyG index in identifying IR in adults.

## 2. Materials and Methods

### 2.1. Study Design

This review was conducted according to the preferred reporting items for systematic reviews and meta-analysis of diagnostic test accuracy studies (PRISMA-DTA statement) [8]. Prior to review conduction, the review protocol was registered in Prospero (Centre for Reviews and Dissemination, University of York) with the access code CRD42018078988.

### 2.2. Eligibility Criteria

Observational, cohort, and cross-sectional studies enrolling adults (18 years or older), with or without type 2 diabetes that evaluated the diagnostic accuracy of the TyG index in identifying IR, compared with any other biochemical marker of IR were included.

We excluded studies with patients <18 years, pregnant women, primary or secondary hypertriglyceridemia, use of medications for hyperlipidemia, malignancy, renal or liver disease, myocardial infarction, and stroke or transient ischemic attacks. There were no exclusion criteria based on language or publications status.

### 2.3. Study Identification

The search strategy was designed and executed by an experienced librarian with input from the principal investigator and the research team. A comprehensive search was conducted in MEDLINE, EMBASE, Web of Science, and Scopus to find eligible studies. All databases were searched from inception to 30 May 2019. MeSH terms, controlled vocabulary, and keywords including the terms “insulin resistance,” “TyG index,” “euglycemic clamp,” and “diagnostic procedure” were combined to search for studies evaluating the TyG index diagnostic accuracy for IR in adults. The reference lists from primary studies and narrative reviews were searched and consulted with experts in the field to obtain any additional references that might have been missed by our initial search strategy. The detailed search strategy is included in [Supplementary-material supplementary-material-1].

### 2.4. Selection of Studies

Two reviewers working independently and in duplicate screened all abstracts and full-text studies for eligibility using the Distiller SR Systematic Review Software (Evidence Partners, Canada). A pilot review was carried out before each phase, and the chance-adjusted agreement was quantified using the kappa statistic (*k* = 0.88). Studies were included when at least one reviewer retrieve. Upon retrieval of potentially eligible studies, the full-text publications were evaluated for eligibility. Disagreements were resolved by consensus. Each reviewer documented reasons for exclusions.

### 2.5. Data Collection Process

Data of the included studies were extracted independently and in duplicate by two reviewers. A standardized data extraction form designed by the authors was used. For the primary outcome, the number of participants, numbers of true positives (TP), false positives (FP), true negatives (TN), and false negatives (FN) were extracted. In addition, the sensitivity and specificity, the 95% confidence intervals (CIs), the overall accuracy, the positive predictive value (PPV = TP/(TP + FP)), the negative predictive value (NPV = TN/(TN + FN)), the positive likelihood ratio (LR+), the negative likelihood ratio (LR−), the diagnostic odds ratio (DOR), and area under the curve (AUC) were extracted. If a study lacked information, if possible, the TP/FP/TN/FN was calculated, and the missing values were computed. Data collected also included type of the study, demographics, study country, TyG index cutoff value, and reference test. Since the formula to calculate the TyG index between authors varied, a linear regression was assessed to obtain a conversion factor between the formula reported by Almeda-Valdés [5] and the rest of the authors ([Supplementary-material supplementary-material-1]). Disagreements were resolved by consensus. If necessary, an expert was consulted to make the final decision.

### 2.6. Risk of Bias of Individual Studies and Quality Assessment

Two review authors worked independently and in duplicate to assess the methodological quality of each study using the Bristol University Risk of Bias Tool, QUADAS-2 [9]. Four key quality domains were assessed: (1) selection of patients; (2) conduction and interpretation of the index test; (3) type and interpretation of the reference standard (considered optimal when it consisted of an euglycemic clamp or the HOMA-IR index); and (4) patient flow, timing, and exclusions.

### 2.7. Summary Measures and Data Synthesis

A narrative synthesis of the included studies was conducted, considering the reference test used and population characteristics. In addition, a comparative summary of the diagnostic accuracy measurements with their confidence intervals is reported.

## 3. Results

### 3.1. Search Results and Study Characteristics

**Figure 1:**
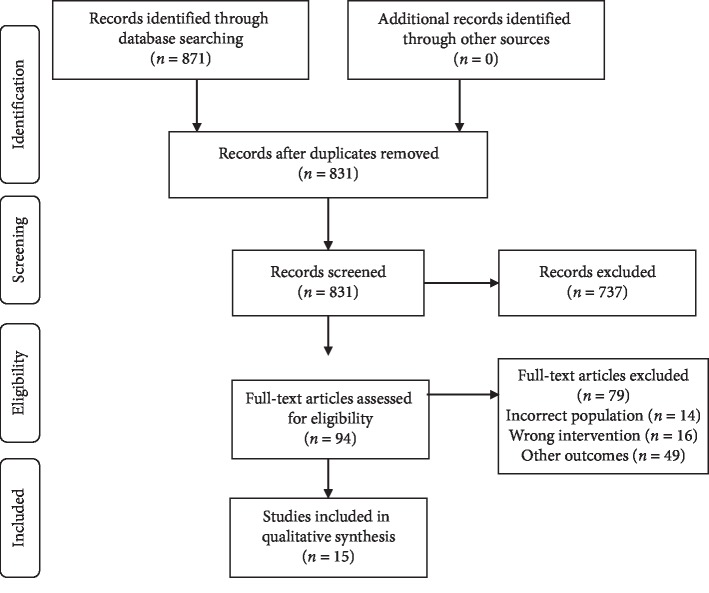
Flowchart of the study selection process.

A total of 871 records were retrieved from which 15 studies enrolling 69,922 participants met the inclusion criteria (Figure 1). The summary of the included studies is presented in Table 1; 14 cross-sectional and one cohort study were included. The included study population comprises healthy controls, insulin-resistant, and type 2 diabetes participants with a high heterogeneity between studies. The reference tests used across studies were the HIEC and the HOMA-IR index. IR was defined with high heterogeneity using different cutoff values across all the studies with both reference tests. The complete details are presented in Table 2.

### 3.2. Diagnostic Accuracy of the TyG Index against HIEC

The TyG index cutoff values of 4.55–5.88 were drawn with a sensitivity >67% and a specificity from 32.5% to 85% in 4 studies with a pooled population of 678 participants using HIEC as the reference test (Table 3) [4, 5, 10–13]. Stratification by gender was available in one study and showed a nonsignificant difference [12]. The AUC was the most consistently reported statistical measure across the studies (0.596–0.858). Positive and negative predictive values were available in 2 studies [5, 12]. Positive likelihood ratios ranged from 1.2 to 6.4, while the negative likelihood ratio ranged from 0.05 to 0.46. The diagnostic odds ratios were estimated with the reported sensitivity and specificity (Table 3) with high variability among studies. Confidence intervals were poorly reported.

### 3.3. Diagnostic Accuracy of the TyG Index against HOMA-IR

In 10 studies, comprising a total of 63,500 subjects, TyG index diagnostic performance was evaluated using the HOMA-IR index as the reference test [7, 12, 14–21]. The cutoff values were reported in 5 (4.55–4.78) with sensitivity and specificity values ranging from 73% to 90% and 45% to 99%, respectively (Table 3). HOMA-2IR was the reference standard in a single study [21]. Different thresholds were used to define IR with HOMA-IR. The thresholds used were derived from previous literature or by participants' percentile values. The AUC values for individual studies ranged from 0.69 to 0.89. The LR+, LR−, and DOR were estimated using the reported sensitivity and specificity described in Table 3.

### 3.4. Risk of Bias

**Figure 2:**
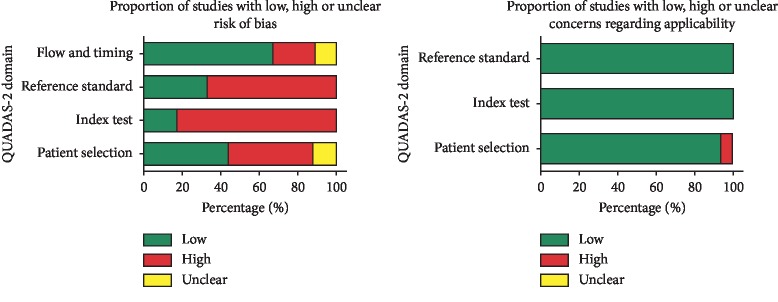
Graphical display summary of the risk of bias judgment using the QUADAS-2 tool.

According to the QUADAS-2 tool, there was an overall moderate to high risk of bias. The patient selection domain was high, unclear, and low risk in eight, one, and six studies, respectively. In the majority of studies, the index test and reference standard domain were at high risk. In 10 studies, we were not able to ascertain whether the reference standard results were known in advance. The flow and timing domain were at the lowest risk due to clear timing description. There was low concern regarding applicability in patient selection, index test, and reference standard domains.  Figure 2 presents a summary of the overall judgment of risk of bias.

## 4. Discussion

### 4.1. Summary of Findings

In this systematic review, we found low-to-moderate quality evidence about the usefulness of the TyG index as a surrogate biochemical marker of IR. Included studies consisted mainly of nondiabetic and middle-aged adults.

HIEC and HOMA-IR were used as the reference standard in the majority of the studies, including a sample of more than 60,000 subjects. Diagnostic accuracy varied according to the reference standard and the definition used to identify IR. In studies using HIEC, diagnostic performance varied with the insulin infusion rate and cutoff value. The highest sensitivity (96%) achieved with a moderate specificity (85%) was found with an insulin M rate of 40 *μ*U/min/m^2^ and a cutoff value of 2.8 insulin mg/min/kg [10]. However, a study with similar characteristics could not reproduce these findings and showed poor sensitivity and specificity. This discrepancy could be explained by a difference in mean age (39.9 ± 9.3 vs. 19.2 ± 1.4) and the inclusion of subjects with diabetes in the first study. Based on the above, the TyG index with a cutoff value of 4.8 has higher sensitivity in young nondiabetic patients.

Studies with HOMA-IR index as a reference standard showed lower diagnostic accuracy measures overall when compared to studies using HIEC. The highest sensitivity and specificity achieved was 90.1% and 99% in the same study, respectively. These studies calculated the HOMA-IR index cutoff value using percentile distribution to identify IR (2.9). Remarkably, all of the studies used different HOMA-IR index cutoff values to define IR, limiting its comparability. Established TyG index cutoff values ranged from 4.55 to 4.78. The majority of the studies used the Youden index to establish the optimal sensitivity and specificity values. Standardized cutoff values could not be calculated due to high heterogeneity between studies.

### 4.2. Comparison with Previous Literature

To date, this is the first systematic review summarizing the diagnostic accuracy of TyG to identify IR in adults. Two similar systematic reviews, focused on the identification of IR in children and adolescents (CRD42018100726) as well as type 2 diabetes prediction (CRD42018114496), are still ongoing according to PROSPERO records. Previous studies have shown a positive association between the TyG index, insulin resistance, and its related conditions. Navarro-Gonzalez et al. [22] found a higher incidence of type 2 diabetes with higher TyG index values. In addition, recent findings demonstrated a positive correlation of the TyG index and the incidence of cardiovascular events providing evidence for a possible association with this metabolic abnormality [23, 24]. The findings of our review do not deny the relationship between IR and the TyG index. However, we found inconsistent results of the TyG index's ability to discriminate between subjects with and without IR. Moreover, the lack of a standardized IR definition limits its clinical utility. Van der Aa Marloes et al. report in a previous systematic review the need to establish well-defined cutoff values and standardized methods of reference tests such as HIEC and HOMA to define IR [25]. In their studies, the definition of IR using HOMA-IR cutoff values ranged from 1.14 to 5.56. In our study, cutoff values had less variation (2–3.5). This difference may be due to the subjects' age differences as the systematic review by Van der Aa Marloes et al. included studies with children.

### 4.3. Implications for Clinical Practice

The TyG index is a noninsulin-based index that is less costly than other insulin based markers. It is accessible from a single sample, which is an advantage for its use in clinical and epidemiological studies. In terms of applicability, glucose and triglycerides are biochemical tests routinely performed in the primary care setting. Hence, the TyG index is an attractive surrogate among lipid ratios for IR detection.

The TyG index has been linked to conditions such as metabolic syndrome, type 2 diabetes, and the risk of developing cardiovascular disease. Based on the above, studies that standardize and evaluate the TyG index capacity as an IR diagnostic marker should be encouraged. However, its applicability is limited due to the marked heterogeneity found in cutoff values and IR definitions among studies. Identifying subjects with IR is fundamental to develop novel treatments and preventive strategies for highly prevalent chronic diseases related to IR, such as obesity and type 2 diabetes [26, 27].

### 4.4. Limitations and Strengths

We acknowledge some limitations. Our results are derived from low-to-moderate quality studies. Likewise, we found different methods for calculating the TyG index due to the mathematical interpretation of its equation. In addition, we could not perform a meta-analysis due to the heterogeneity found in IR definition. We provide the conversion factor to give clarity and facilitate comparison between scales ([Supplementary-material supplementary-material-1]). In counterpart, the review is strengthened by a comprehensive research strategy and enhanced by the simultaneous and rigorous conduction by two reviewers to perform the complete review process.

## 5. Conclusions

In this systematic review, we found moderate-to-low quality evidence about the usefulness of the TyG index as a surrogate biochemical marker of IR. Although there is an association between TyG and IR, due to the lack of a standardized IR definition and high heterogeneity between studies, further validation and standardized cutoff values are needed to be used in clinical practice.

## Figures and Tables

**Table 1:** Study characteristics according to the reference standard of IR.

Reference	Design	Country	Population	Age^*∗*^	TyG, *n*	Ref. Study, *n*
*Hyperinsulinemic-euglycemic clamp*					6422	678
Guerrerro-Romero, [10]	Cross sectional	Mexico	Nondiabetic and diabetic adults	39.9 ± 9.3	99	99
Vasques, [11]	Cross sectional	Brasil	Nondiabetic and diabetic adults	47.3 ± 14.6	82	82
Bastard, [4]	Cohort study	France	Overweight postmenopausal women	57.3 ± 0.4	163	163
Guerrerro-Romero, [12]	Cross sectional	Mexico	Nondiabetic young adults	19.2 ± 1.4	5538	75
Qu, [13]	Cross sectional	China	Mixed population^*∗*^^*∗*^	Control 27 ± 4, 59 ± 10, PCOS 28 ± 6, IGT 59 ± 10, T2DM 58 ± 9	483	202
Almeda-Valdés, [5]	Cross sectional	Mexico	Nondiabetic adults	32.9 ± 11	57	57

*HOMA-IR*					63,500	63,500
Simental-Mendía, [7]	Cross sectional	Mexico	Nondiabetic adults	41.4 ± 11.2	748	748
Du, [14]	Cross sectional	China	Nondiabetic adults	50.6 (39.3–60.7)M, 51 (40.3–60.6)F	7629	7629
Lee, [15]	Cross sectional	Korea	Nondiabetic adults	42.4 ± 0.3M, 44.1 ± 0.3F	17029	17029
Er, [16]	Cross sectional	Taiwan	Nondiabetic adults	43 (38.1–50)M, 46 (40–51.2)F	511	511
Guerrero-Romero, [12]	Cross sectional	Mexico	Nondiabetic adults	19.2 ± 1.4	5538	5538
Mazidi, [17]	Cross sectional	China	General population	47.6	18318	18318
Thota, [18]	Cross sectional	Australia	Nondiabetic elderly adults	77.78 ± 7.16	486	486
Lim, [19]	Cross sectional	Korea	Nondiabetic adults	45.2 ± 15.0M, 44.3 ± 14.6F	11149	11149
Dorota-Łojko, [20]	Cross sectional	Poland	Nondiabetic and diabetic adults with bipolar disorder	58.1 ± 11.7	88	88

*Other surrogates*					2004	2004
Salazar, [21]	Cross sectional	Venezuela	Nondiabetic adults	39.6 ± 15.3	2004	2004

^*∗*^Median ± SD or median values. ^*∗*^^*∗*^Healthy controls, polycystic ovarian syndrome, type 2 diabetes mellitus, and obese women. M: male; F: female; PCOS: polycystic ovarian syndrome; IGT: impaired glucose tolerance; T2DM: type 2 diabetes mellitus.

**Table 2:** Summary of the reference test.

Study	Reference test	IR definition	IR cutoff value
Guerrero-Romero, [10]	HIEC	M rate (insulin 40 *μ*U/min/m^2^)	2.8 insulin 40 mg/min/kg
Junqueira-Vasques, [11]	HC	NR	NR
Bastard, [4]	HIEC	M rate (insulin 75 *μ*U/min/m^2^)	11.56 mg/min/kgFFM
Guerrero-Romero, [12]	HIEC	M rate (insulin 40 *μ*U/min/m^2^)	2.8 insulin mg/min/kg
Qu, [13]	HIEC	M rate (insulin 1 mU/kg/min)	6.28 mg/min/kg
Almeda-Valdés, [5]	HIEC	M rate (insulin 50 mU/min/m^2^)	6.39 mg/min/kgFFM
Simental-Mendía, [7]	HOMA-IR	Previous literature	≥2.8
Du, [14]	HOMA-IR	>75th percentile	3.5
Lee, [15]	HOMA-IR	>75th percentile	2.52
Er, [16]	HOMA-IR	>75th percentile	2.43
Guerrero-Romero, [12]	HOMA-IR	Not specified	≥2.9
Mazidi, [17]	HOMA-IR	Not specified	≥2.5
Thota, [18]	HOMA-IR	Not specified	NR
Lim, [19]	HOMA-IR	>75th percentile	NR
Dorota-Łojko, [20]	HOMA-IR	Previous literature	≥2.0
Salazar, [21]	HOMA-2IR	≥2.0	≥2.0

IR: insulin resistance; HC: hyperglycemic clamp; HIEC: hyperinsulinemic-euglycemic clamp; FFM: free-fat mass; NR: not reported; M = male; *F* = female; PreF = premenopausal female; PF = postmenopausal female.

**Table 3:** Summary of the diagnostic accuracy measures reported for the TyG index.

Study	Reference	TyG cutoff	Sensitivity (%)	Specificity (%)	PPV (%)	NPV (%)	PLR	NLR	DOR	AUC
Guerrero-Romero, [10]	HIEC	4.68	96	85	NR	NR	6.4	0.05	136	0.85
Guerrero-Romero, [12]	HIEC	4.68M	67M	72M	38M	90M	2.4M	0.45M	5.2M	0.67M
		4.55F	68F	66F	44F	84F	2.04F	0.48F	4.1F	0.68F
Qu, [13]	HIEC	4.55	67	72	NR	NR	2.4	0.46	5.22	0.77
Almeda-Valdés, [5]	HIEC	4.43	85.7	32.5	29.2	87.5	1.2	0.45	2.88	0.59
Simental-Mendía, [7]	HOMA-IR	4.65	84	45	81	84	1.5	0.36	4.29	NR
Guerrero-Romero, [12]	HOMA-IR	4.68M	90.9	99.7	98.3	98.6	NR	0.09	3319.6	NR
		4.55F								
Mazidi, [17]	HOMA-IR	4.78	75.9	71.9	NR	NR	2.7	0.34	8.05	0.81
Dorota-Łojko, [20]	HOMA-IR	4.69	73.8	75.6	NR	NR	3.0	0.35	8.72	0.78
Salazar, [21]	HOMA-2IR	4.49	82.6	82.1	NR	NR	4.6	0.21	21.77	0.88

M: male; F: female; HC: hyperglycemic clamp; HIEC: hyperinsulinemic-euglycemic clamp; TyG: triglyceride/glucose index; PPV: positive predictive value; NPV: negative predictive value; PLR: positive likelihood ratio; NLR: negative likelihood ratio; DOR: diagnostic odds ratio; AUC: area under the curve; NR: not reported.

## Supplementary Materials

Supplementary Materials Appendix A: search strategy for MEDLINE, EMBASE, Web of Science, and Scopus. Appendix B: conversion factor between scales of the TyG index. .
