# Supplementary material for: Diagnostic Accuracy of the Triglyceride and Glucose Index for Insulin Resistance: A Systematic Review
Source: Int J Endocrinol. 2020 Mar 10;2020:4678526. doi: 10.1155/2020/4678526 (PMC7085845; doi:10.1155/2020/4678526)
Supplement: Supplementary Materials — Appendix A: search strategy for MEDLINE, EMBASE, Web of Science, and Scopus. Appendix B: conversion factor between scales of the TyG index. . [file 4678526.f1.docx]

**Appendix A.** Search Strategy for MEDLINE, EMBASE, Web of Science, and Scopus.

Complete strategy (Copy and paste):

((("euglycaemic clamp" or "euglycaemic clamping" or "euglycemic clamp" or "Glucose Clamp" or "Glucose Clamp Technic" or "Clamp Euglycaemic" or "Clamp Euglycemic" or "Clamp Glucose" or "Clamping Euglycaemic" or "Clamping Euglycemic" or "Clamping Glucose" or "Clamps Euglycaemic" or "Clamps Euglycemic" or "Clamps Glucose" or "Euglycaemic Clamps" or "Euglycemic Clamps" or "Glucose Clamp Technics" or "Glucose Clamp Techniques" or "Glucose Clamps" or "Technic Glucose Clamp" or "Technics Glucose Clamp" or "Technique Glucose Clamp" or "Techniques Glucose Clamp" or "Euglycemic Clamping" or "Glucose Clamping" OR "Intravenous Glucose" or "Tolerance Intravenous Glucose" or "Tolerance Test" or "OGTT" or "Oral Glucose Tolerance" or "Oral Glucose Tolerance Test" or "Glucose Tolerance Tests" or "Glucose Tolerance Oral" or "fasting glucose" or "fasting glucose level*" or ("fasting glucose" w/3 "level*") OR("fasting insulin" or "fasting insulin level*") or ("fasting insulin" w/3 "level*") OR ("glucose-insulin ratio") or ("glucose-insulin" w/3 "ratio") or "insulin-glucose ratio" or ("insulin-glucose" w/3 ratio) or "glucose-insulin product" or ("glucose-insulin" w/3 "product") or "insulin-glucose product" or ("insulin-glucose" w/3 "product") OR ("HOMA" or "homeostatic assessment model" or "homeostasis model of assessment of insulin resistance" or "HOMA-IR" or "HOMA*") OR ("quantitative insulin sensitivity check index" or "disease assessment" or ALL("quicki")) OR ("matsuda" or "matsuda index" or ("matsuda" w/3 "index")) OR (insulinogenic or "insulinogenic index" or (insulinogenic w/3 index)))) AND ((ALL(insulin resistance or insulin resistance or insulin sensitivity or insulin sensitivity or resistance insulin or resistance insulin or sensitivity insulin or sensitivity insulin))) AND ((ALL(triacylglycerol or triacylglycerol blood level or triacylglycerol level)) AND (ALL(glucose))) AND (diagnostic procedure or medical procedures or analysis or "assessment of humans" or blood examination or clinical assessment tool or diagnosis or endocrine system examination or examination or measurement or screening)) OR ("TyG index" or "triglyceride-glucose index" or ("triglyceride-glucose" w/2 "index") or ("glucose-triglyceride index") or ("glucose-triglyceride" w/2 "index"))

**Appendix B.** Conversion factor between scales of TyG index.

**TyG index^a^ = [0.34+0.5(TyG index^b^)] ± (SDx0.5)**

^a^: TyG = Ln (Triglycerides [mg/ml] * Fasting plasmatic Glucose [mg/ml]) /(2)

^b^: TyG= (Triglycerides [mg/dl]*Fasting plasmatic Glucose[mg/dl])/(2)
